# Colonization of Abandoned Land by *Juniperus thurifera* Is Mediated by the Interaction of a Diverse Dispersal Assemblage and Environmental Heterogeneity

**DOI:** 10.1371/journal.pone.0046993

**Published:** 2012-10-10

**Authors:** Gema Escribano-Avila, Virginia Sanz-Pérez, Beatriz Pías, Emilio Virgós, Adrián Escudero, Fernando Valladares

**Affiliations:** 1 Departamento de Biología y Geología, Universidad Rey Juan Carlos, Madrid, Spain; 2 Facultad de Ciencias Ambientales, Universidad de Castilla-La Mancha, Toledo, Spain; 3 Departamento de Biología Vegetal I, Universidad Complutense de Madrid, Madrid, Spain; 4 Museo Nacional de Ciencias Naturales, Consejo Superior de Investigaciones Científicas, Madrid, Spain; Institut Mediterrani d’Estudis Avançats (CSIC/UIB), SPAIN

## Abstract

Land abandonment is one of the most powerful global change drivers in developed countries where recent rural exodus has been the norm. Abandonment of traditional land use practices has permitted the colonization of these areas by shrub and tree species. For fleshy fruited species the colonization of new areas is determined by the dispersal assemblage composition and abundance. In this study we showed how the relative contribution to the dispersal process by each animal species is modulated by the environmental heterogeneity and ecosystem structure. This complex interaction caused differential patterns on the seed dispersal in both, landscape patches in which the process of colonization is acting nowadays and mature woodlands of *Juniperus thurifera*, a relict tree distributed in the western Mediterranean Basin. Thrushes (*Turdus* spp) and carnivores (red fox and stone marten) dispersed a high amount of seeds while rabbits and sheeps only a tiny fraction. Thrushes dispersed a significant amount of seeds in new colonization areas, however they were limited by the presence of high perches with big crop size. While carnivores dispersed seeds to all studied habitats, even in those patches where no trees of *J. thurifera* were present, turning out to be critical for primary colonization. The presence of *Pinus* and *Quercus* was related to a reduced consumption *of J. thurifera* seeds while the presence of fleshy fruited shrubs was related with higher content of *J. thurifera* seeds in dispersers’ faeces. Therefore environmental heterogeneity and ecosystem structure had a great influence on dispersers feeding behaviour, and should be considered in order to accurately describe the role of seed dispersal in ecological process, such as regeneration and colonization. *J. thurifera* expansion is not seed limited thanks to its diverse dispersal community, hence the conservation of all dispersers in an ecosystem enhance ecosystems services and resilience.

## Introduction

Ecosystems are changing at an unprecedented rate in response to global change [1]. One of its most powerful and probably least studied drivers is land use change. During the last century two opposing forces have coexisted in well-developed regions, such as at the northern fringe of the Mediterranean basin [2], regarding land use: either intensification or abandonment [3], [4]. Abandonment is currently occurring in low productive areas (e.g. difficult accessible slopes, steep mountain areas) where the rural exodus has been very significant [5]. Current vegetation dynamics in abandoned fields is modulated by factors such as, past use and management history, soil characteristics, climate and propagules availability [6]. The arrival of seeds to non-forested areas is a key stage in the process of colonization of abandoned agricultural lands [7] as seed dispersal decreases with the distance to the forest edge [8].

For fleshy fruited species the arrival of seeds to non-forested areas is a function of the abundance, composition and behaviour of the members of the dispersers’ community [9]. The service provided by each disperser to a given plant species varies according to differences in both, the quantitative and qualitative components of the dispersal process [10]. The quantitative components are related to differences in the number of visits to a feeding plant, fruits dispersed per visit and local abundance of dispersers. While qualitative component would be mediated by differences in seed retention time, gut treatment and movement behaviours such as home *versus* foraging range and daily movement patterns (e.g. scent marking, anti-predator behaviour) [11]. Despite dispersers differ in these dispersal components [12], [13], [14], most of the research on seed dispersal mutualisms has been focused on single species, or at the best, in single functional groups. However the few works in which the complete assemblage was considered have reinforced the idea that each species differentially contributed to the quantitative and qualitative terms of the dispersal process [12], [15], [16], [17]. In order to assess how seed dispersal contributes to critical ecological processes such as forest maintenance, regeneration and colonization, the complete community of potential dispersers and their differential behaviour need to be taken into account [16], [18], [19]. Composition of the dispersers’ community and their behavior could vary according to the environmental heterogeneity and ecosystem structure (e.g. woody and shrub cover, fruiting environment) [19], [20]. This knowledge seems critical in order to unveil how woodland expansion due to land abandonment operates and to develop adequate management strategies.

Woodlands of *Juniperus thurifera* have been subjected to a traditional management (e.g. logging, grazing and destruction for crop cultivation), however since the middle of the XIX century these activities have drastically decreased allowing the species to increase in density and currently to colonize abandoned fields [21], [22] which is provoking a spectacular shift into new colonization areas [23]. *J. thurifera* is a fleshy fruited relict tree with a diverse assemblage of legitimate seed dispersers, such as thrushes *Turdus* spp. [24], [25]; carnivores, such as red foxes (*Vulpes vulpes)* and stone martens (*Martes foina)* [12], [15]; herbivores, as rabbits (*Oryctolagus cuniculus*) [12], [26] and even domestic sheeps (*Ovis aries)* [27]. Since all these animals profoundly differ in their quantitative and qualitative efficiency on the dispersal process [10], their relative contribution to the expansion process of *J. thurifera* must be markedly different.

Dispersal season of *J. thurifera* occurs from the middle autumn until the end of the winter, during this period carnivores, thrushes and rabbits are active frugivores when other food resources are scare [12], [24], [25], [26], [27], [28] hence we expect all of them will contribute to increase the density of mature woodlands by dispersing high quantity of seeds. Our expectations are that in new colonization areas, where isolated junipers remained, carnivores and thrushes should be the main dispersers, as they can transport a relatively large quantity of seeds [16]. However, the contribution of thrushes, as specialist feeders, will be conditioned to the presence of fruiting trees and crop size. In addition, we expect that the arrival of seeds to remaining active agricultural lands, where *J. thurifera* is absent, will be carried out mainly by carnivores according to their generalist diet and wide home range [19], [28]. We also expect that sheeps disperse a low amount of seeds being their contribution to the dispersal process marginal [12]. Dispersers’ post-feeding behaviour that conditioned the deposition pattern is also critical. In this sense we hypothesized that thrushes will disperse more seeds beneath the canopy of adult junipers or other fleshy fruited species. Rabbits will disperse more seeds on open pasture where they feed and under the canopy of fruiting trees where warrens used to be located. Carnivores, according to their territoriality and scent marking, will disperse more seeds to visible and conspicuous non canopied microhabitats.

In order to test our hypothesis we evaluate the role of seed dispersal in the expansion process of *J. thurifera* woodlands into new colonization areas by considering the whole dispersal assemblage community, their feeding behaviour and dispersal deposition pattern and how environmental heterogeneity occurring in the ecosystems influence dispersers behaviour. Specifically, we evaluate the following questions: a) what is the quantitative contribution of each member of the dispersal assemblage community to the seed dispersal in the different habitats: mature woodland, new colonization areas and active agricultural lands? b) How the deposition patterns of each disperser condition the arrival or seeds to different microhabitats? c) Are the quantitative contribution of each disperser and their seed deposition patterns consistent across different sites?

## Materials and Methods

### Ethics Statement

All necessary permits were obtained for the described field studies from the Dirección General de Montes y Espacios Naturales de Castilla-La Mancha. All animal work was conducted according to relevant Spanish and international guidelines.

### Study Area

**Figure 1:**
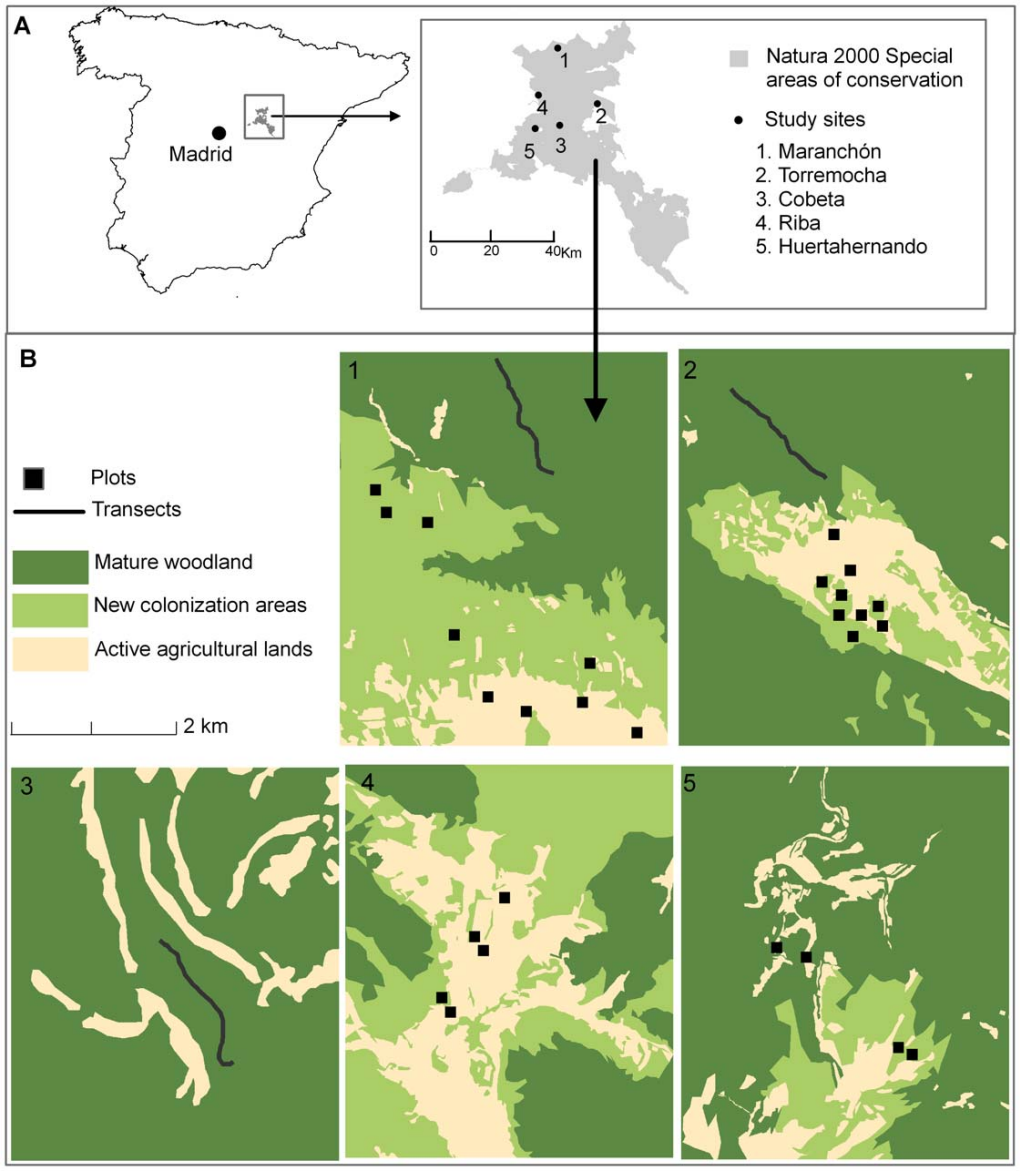
Location, study area and sampling design. Fig. 1A, on the top left of the figure we present the location of the study area within the Iberian Peninsula. On the top right, a zoom to the protected areas, Alto Tajo and Parameras de Maranchón, Hoz de Mesa y Arangocillo (Natural 2000 Network) where the study sites are located. Fig.1B, a graphical description of the different study sites and the amount and distribution of the habitats describing the ongoing process of *J. thurifera* expansion.

The study was conducted during *J. thurifera* dispersal season 2008–2009 in *Alto Tajo* and *Parameras de Maranchón, Hoz de Mesa y Arangocillo,* both of them Special Areas of Conservation of the Natura 2000 network located in Guadalajara province, central Spain. The study area covers 40 km^2^ (centroid 40° 55¢ N, 2° 10¢ W) (Figure 1A).The climate is Mediterranean continental with rainfall around 500 mm per year with a pronounced summer drought. Mean annual temperature is 10.2°C, with January being the coldest month (mean temperature: 2.4°C) and July the warmest (mean temperature: 19.5°C). Snowfalls occur from November to April (www.aemet.es).The mean elevation of the area is 1200 m where the vegetation is mainly composed by open woodlands dominated by *J. thurifera.*

### Sampling Design

The complexity of the territory was classified in three habitats which describe the ongoing process of expansion, mature woodland remnants (MW), new colonization areas (NCA) and ongoing active agricultural lands (AL). *J. thurifera* cover on MWs was over 30% with a high abundance of adult trees. NCAs were abandoned agricultural fields or livestock pastures patches where *J. thurifera* cover was under 15%, being most of the individuals newcomers. In order to evaluate if the expansion process was limited to areas with some *J. thurifera* remanent or contrary if patches without any individual of the species could receive seeds, AL without any *J. thurifera* tree were also included in the study. These habitats did not suffer any management tasks during the survey process. We selected different sites in which the habitats MW, NCA and AL were represented: Maranchón, Torremocha, Cobeta, Riba and Huertahernando (Figure 1A). All habitats were represented in Maranchón and Torremocha, in Cobeta only MW was found while in Riba and Huertahernando only NCA and AL habitats were studied (Figure 1B). In each MW, we selected a total area of 2000×50 m in which seed dispersal process was studied independently for each functional group of dispersers (see Faeces collection below). In NCA and AL seed dispersal was studied in plots of 100×50 meters. A total of 16 NCA plots were selected which were unevenly distributed among Maranchón (6 plots), Torremocha (5), Riba (3) and Huertahernando (2) as a function of the available habitat fragments. For AL a total of 12 plots were selected distributed among Maranchón (4 plots), Torremocha (4), Riba (2) and Huertahernando (2) (Figure 1B).

### Faeces Collection

Thrush pellets, carnivore scats and rabbit and sheep droppings (discrete clumps containing from 15 to 20 pellets) were considered as individual and comparable faecal deposits (hereafter faeces). Faeces collection was conducted during the dispersal season (December-March). Each faeces was collected and packed in a paper bag and the microhabitat (*J. thurifera*, shrub or open area) in which each faeces was found recorded. We considered the influence area of *J. thurifera* and shrub microhabitats of 6 and 4 meters respectively away from the trunk. When a faeces was found within a further distance of these microhabitats it was considered as an open microhabitat.

The survey procedure differs between the studied habitats according to the detectability of the dispersers in each habitat and species behaviour. MWs consisted on homogeneous and continuous areas in the territory with a high percentage of tree cover and fruiting trees, therefore dispersers will highly occupied these habitats [12]. Whereas NCAs-ALs consisted on discrete and small fragments with a reduced tree cover and fruits availability. Thus, a reduced occupancy in relation MW is expected. A reduced occupancy is related with a lower detectability [29], [30], [31], [32], therefore in order to obtain reliable data about the occupancy of all studied habitats the sampling effort cannot be identical in areas of large occupancy (MW) and those with sporadic or lower intensity of occupancy (NCA and AL). Hence, more sampling effort was needed in those areas where the occupancy and thus detectability is lower. According to this, and attending to different behaviour and movement patterns of dispersers we performed a different sampling scheme for thrushes and mammals. Thrushes dispersal was assessed twice along the study period, coinciding with the moment of thrushes’ censuses (see thrushes’ abundance section*)*. Thrushes perform a non-random use of the habitat being quite focused to trees with large fruit availability and high size for perching [12], [24]. Therefore we performed a stratified sampling focused on trees in mature woodland where the cover of trees was high. We sampled 15 sub-plots within the area selected in each MW. We sampled 10 transects of 1×10 m located at random compass direction away from the microhabitats *J. thurifera tree* and shrub [12] in each sub-plot. However, in NCA very few trees for perching were available and trees with cones were usually one while in AL they were totally absent. In order to avoid overestimation of seed dispersal in NCA and AL patches the total surface of the plot of those habitats was sampled. Mammal’s dispersal was assessed fortnightly during the whole dispersal season. Faeces in each MW were collected in a 2000×3 m transect within the selected area, this methodology is optimum for areas with high occupancy and detectability [31]. However, according to the lower detectability on NCA and AL, we needed to increase the sampling effort, therefore we sampled the whole surface of the plot of the mentioned habitats. All fresh faeces were collected and the microhabitat in which they occurred, according to the criteria defined above, recorded.

### *Juniperus Thurifera* Crop Size

We randomly selected 20 adult fruiting trees in each MW and 1 in each NCA (rarely more than one was present). In each tree we counted all the arcestides (organs equivalent to fleshy fruits) inside 4 quadrates of 15×20 cm randomly located in the crown at different heights and compass directions. Since we used a density estimate of arcestides as surrogate of crop size, the sum of the counted arcestides in the four samples was divided by the total sampled area for each tree.

### Tree and Shrub Description

We identified all tree and woody shrub species and their covers in all sites. In each MW we walked along 2 km lineal transect. Every 100 meters we established a 15 meter meters radius circumference in which the percentage cover of each tree species and woody shrubs was estimated. The final cover was the result of add up the twenty partial percentage covers for each species. In the case of NCA plots all trees and woody shrubs present were identified and their percentage cover established.

### Thrushes’ Abundance

We conducted two thrushes’ censuses during the study period, in November 2008 (early winter) and in February 2009 (late winter). Within the area selected in MW we established a 2 km length transect with a main belt 50 m wide [12]. In NCA and AL the census were undertaken from a watching point in the centre of each plot. All thrushes seen or heard, walking along the transects or in the watching points were recorded. Total observation effort was 20 hours, 6 in MW, 8 in NCA and 6 in AL. We started census at sunrise and stopped at 11∶00 hours. Both early and late winter censuses were conducted during three consecutive days with favourable weather by the same two observers.

### Data Analyses

Variation in crop size was analysed using a general mixed model with the number of arcestides (log transformed for obtaining normal error distributions) as response variable with, habitat (MW, NCA) as fixed factor and site as random factor. In order to evaluate the possible effects of environmental heterogeneity (total cover of tree species different to *J. thurifera* and total cover of fleshy fruited species) in dispersers feeding behaviour we performed two Generalized Linear Mixed models (GLMMs). To test if environmental heterogeneity influenced the choice of dispersers to consume or not consume *J. thurifera* seeds we used the presence/absence of *J. thurifera* seeds in each faeces as response variable with Binomial error distributions and logit as link function. To test if the environmental heterogeneity influenced the quantity of *J. thurifera seeds* consumed by disperses we used the total number of *J. thurifera* seeds dispersed in each faeces as response variable with Poisson error distribution and log as link function. In both models habitat and disperser were used as fixed factors and the variables defining environmental heterogeneity: total cover of tree species different to *J. thurifera* and total cover of fleshy fruited species different of *J. thurifera*, were used as additional fixed factors maintaining site as random factor. In order to test for differences in seed dispersal according to site, habitat, microhabitat and disperser we performed a new GLMM with the density of dispersed seeds per hectare as response variable and habitat, microhabitat and disperser as fixed factors together with site as a random factor being the corresponding error distribution Gaussian and the link function identity. Active agricultural lands habitats (AL) were analysed separately according to the lack of covered microhabitats. In this case a GLMM was performed with density of dispersed seeds per hectare as response variable, disperser as fixed factor and site as random factor with a Gaussian error type and the link function identity. All analyses were conducted in R environment [33] with additional packages “nlme” [34] and “lme4” [35].

## Results

### Tree and Shrub Description

Maranchón MW had the lowest tree cover among juniper woodlands having Cobeta the highest. In NCAs, Huerta and Torremocha were more open than Maranchón and Riba, which presented the highest cover (72%). The most common fleshy fruited species apart from *J. thurifera* was *J. communis* except in Riba where this rank position was occupied by *Rosa* spp (11.7%) and *J. oxycedrus* (4%). On the other hand *J. phoenicea* (2.3%) was present only in Cobeta which was the MW with the highest number of tree species with more than a tenth percentage covered by pines and oaks (Table 1).

**Table 1:** Tree and shrub species cover.

Site	Habitat	Cover	JT	JC	JO	JP	R	GS	PH	QI	QF
Maranchón	MW	42	30.6	10.2			0.8	1.2			
	NCA	28	10	8.1			0.9	9.3			0.4
Torremocha	MW	60	46	6.6			0.1	9.7		2.5	
	NCA	21	8	0.8			1	10.6		0.1	0.9
Cobeta	MW	71	47	5.7	2.1	2.3	0.3	1.8	17.6	10.8	0.7
Riba	NCA	72	4	0.2	4		11.7	60			0.4
Huertahernando	NCA	10	10				1	1		1	

MW: mature woodland; NCA: new colonization areas; Cover: Total percentage cover (%); Percentage cover of the species (%): JT: *Juniperus thurifera*; JC: *Juniperus communis*; JO: *Juniperus oxycedrus*; JP: *Juniperus phoenicea*; R: *Rosa spp*; GS: *Genista scorpius*; PH: *Pinus halepensis*, QI: *Quercus ilex*; QF: *Quercus faginea*. Blank space indicates the species was not present.

### *Juniperus Thurifera* Crop Size

**Figure 2:**
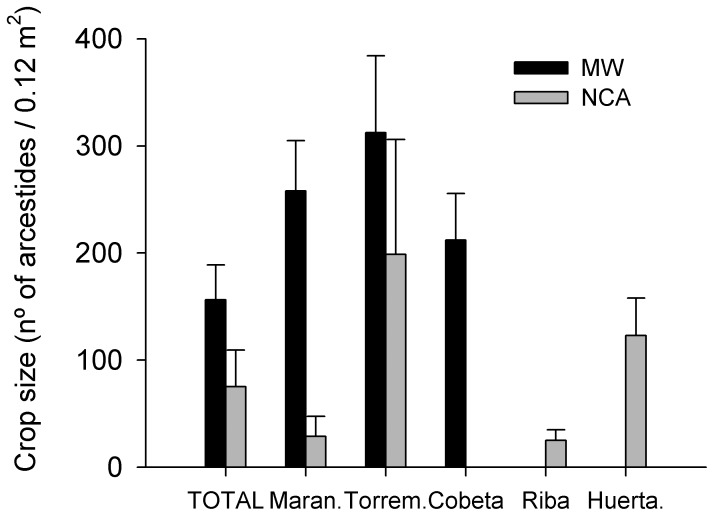
Crop size. Crop size (mean ± SE number of arcestides*0.12m^−2^) on mature woodlands (MW) and new colonization areas (NCA) for all studied sites. TOTAL (mean ± SE number of arcestides/0.12m^2^) for each habitat type is represented on the first two columns.

Arcestides density differed significantly between habitats MWs had a higher density of arcestides than NCA. The estimates were 2.02, 1.37 for MW and NCA respectively which were significantly different from 0 (P value <0.001) in both cases. Arcestides density was similar for the MWs of Torremocha, Maranchón and Cobeta (Figure 2).

### Thrushes’ Abundance

A total of five species of thrushes were recorded during the censuses: *Turdus viscivorus* (57.1% of thrushes), *Turdus philomelos* (35.8%), *Turdus merula* (4.3%), *Turdus iliacus*(2.1%), and *Turdus pilaris* (0.7%). In general thrushes’ abundance was higher in MW than in NCA and AL. However in Torremocha and Riba thrushes’ abundance in NCA was similar to MW during the late winter. A similar abundance was found for the three MWs in the two dates, though a decreased was found in Cobeta in late winter. In general we did not observe thrushes in AL with the exception of Torremocha (Table 2).

**Table 2:** Thrushes abundance.

Site	Habitat	Early Winter	LateWinter
Maranchón	MW	9.1	13.8
	NCA	1.7	0.33
	AL	0	0
Torremocha	MW	11.4	9.1
	NCA	0	12.8
	AL	0.5	7.5
Cobeta	MW	12.3	6.4
Riba	NCA	2	12
	AL	0	0
Huertahernando	NCA	0	0
	AL	0	0

Thrushes’ abundance (*Turdus* spp* Ha^−1^) in the three habitats, MW: mature woodland; NCA, new colonization areas; AL, active agricultural lands.

### Environmental Heterogeneity, Faeces Abundance and Number of *J. thurifera* Seeds Per Faeces

A total of 1627 faeces were collected during the study period, of which the highest number were thrushes’ pellets (1192), then herbivores droppings (224) and lastly carnivores (211). One hundred faeces were collected in AL and therefore were not used for the analysis of dispersers feeding behaviour and environmental heterogeneity analyses due to the lack of natural vegetation in these plots.

Stone marten was the species which dispersed the higher number of faeces with presence of *J. thurifera* and sheeps the lowest. Tree cover excluding *J. thurifera* was negatively related to the presence of *J. thurifera* seeds on dispersers faeces but not to the cover of fleshy fruited species different to *J. thurifera* (Table 3). The number of seeds contained in each faeces was opposite to the rank of number of faeces: carnivores dispersed the highest number of seeds per faeces (36 in average), then herbivores (4) and lastly thrushes (0.7). In general both variables, the number of faeces and *J. thurifera* seed per faeces decreased from the MW to AL for all dispersers (Table 4 and Table S1). Tree cover excluding *J. thurifera* was also negatively related to the number of seeds of *J. thurifera* dispersed per faeces while total cover of fleshy fruited species apart from *J. thurifera* was positively related (Table 4).

**Table 3:** General linear model result for *J. thurifera* seeds presence/absence in each faeces.

Fixed effects		Parameter value	SE	z value	Pr(>|z|)
Intercept		0.31	1.56	0.20	0.842
**Disperser**	**Stone marten**	**1.46**	**0.56**	**2.61**	**0.009**
	Red fox	0.69	0.50	1.39	0.166
	Thrushes	0.46	0.45	1.02	0.309
	**Sheep**	**−1.64**	**0.49**	**−3.32**	**0.001**
**Habitat**	**New colonization areas**	**−0.67**	**0.26**	**−2.58**	**0.010**
Fruits cover		0.03	0.05	0.66	0.510
**Tree cover**		**−0.45**	**0.10**	**−4.26**	0.000
**Random effects**					
	Intercept	Residual			
SD	9.67	3.11			

Significant effects (P<0.05) are indicated in bold. When P value was smaller than 0.001, <0.001 was indicated. D.F: degrees of freedom. SE: Standard Error. SD: Standard Deviation. Missing levels of factors (disperser: rabbit; habitat: woodland) are included on the intercept.

**Table 4:** General linear model result for *J. thurifera* seeds abundance per faeces.

Fixed effects		Parameter value	SE	z value	Pr(>|z|)
**Intercept**		**2.62**	**1.18**	**2.23**	**0.03**
**Disperser**	**Red fox**	**1.05**	**0.06**	**18.21**	**<0.001**
	**Stone marten**	**0.94**	**0.06**	**15.95**	**<0.001**
	**Sheep**	**−2.33**	**0.10**	**−24.01**	**<0.001**
	**Thrushes**	**−3.13**	**0.07**	**−47.15**	**<0.001**
**Habitat**	**New colonization areas**	**−1.81**	**0.09**	**−20.16**	**<0.001**
**Fruits cover**		**0.06**	**0.02**	**2.64**	**0.008**
**Tree cover**		**−0.40**	**0.05**	**−8.68**	**<0.001**
**Random effects**					
	Intercept	Residual			
SD	6.61	2.57			

Significant effects (P<0.05) are indicated in bold. When P value was smaller than 0.001, <0.001 was indicated. D.F: degrees of freedom. SE: Standard Error. SD: Standard Deviation. Missing levels of factors (disperser: rabbit; habitat: woodland) are included on the intercept.

In relation to deposition patterns in the MWs, the role of thrushes was similar in the three sites and they usually preferred *J. thurifera* canopied microhabitats. Herbivores, especially rabbits, deposited more faeces and a higher number of seeds per faeces in Torremocha than in the other sites and mainly in open microhabitats. Most carnivores faeces collected contained *J. thurifera* seeds in the MW of Maranchón (84%) and Torremocha (94%) with a high number of seed per faeces (67 and 96 respectively). By contrast, in the MW of Cobeta most carnivores faeces contained small-mammals remnants and seeds of two coexisting congeners, *Juniperus oxycedrus* and *Juniperus phoenicea* being the average number of *J. thurifera* seeds per faeces really low (5). The microhabitat preferred by carnivores in MW was shrub (Table S1).

Deposition patterns in NCA patches of Maranchón and Torremocha were quite similar to those on the MW but with a lower abundance of faeces and seeds per faeces. In Riba we found that carnivores, mainly stone martens, disperse a high number of faeces in comparison with the rest of the sites. It is worth noting that faeces did not contain any seed of *J. thurifera* in this last case, while congener *J. oxycedrus* seeds were abundant. A similar pattern in the number of deposited faeces *versus* dispersed seeds per faeces was found for thrushes which deposited the highest number of faeces of all NCAs in Riba although only 15% of them contained *J. thurifera* seeds. They had a low number of seed per faeces (0.21), being *Rosa* spp seeds abundant. We did not found any carnivores’ faeces in Huerta and only thrushes generated some seed dispersion preferring the *J. thurifera* canopied microhabitat (Table S1).

In AL the total number of faeces and seed number per faeces sharply decreased in relation to MW and NCA. The dispersal pattern generated by dispersers was site depended. Thus in Maranchón only foxes produced dispersal patterns, in Torremocha both species of carnivores, sheeps and thrushes deposited faeces and seeds while in Huerta only foxes and thrushes did. Finally we did not found any dispersed seed by any disperser in the Riba AL (Table S1).

### Seed Dispersal

**Figure 3:**
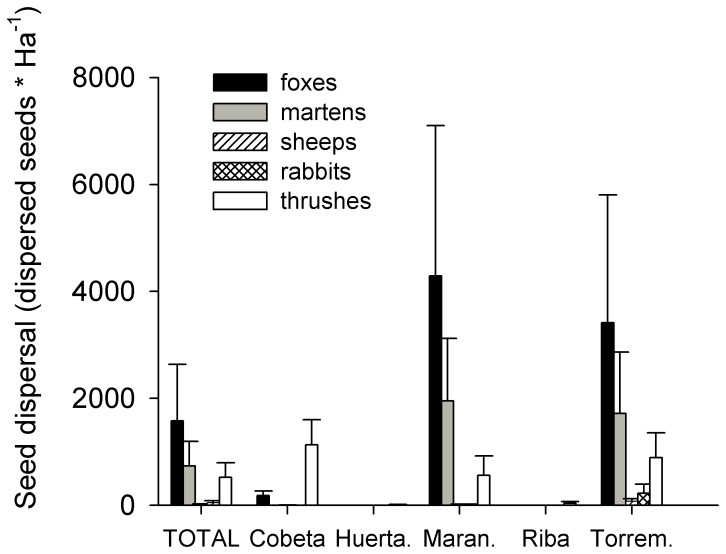
Seed dispersal site and dispersers. Seed dispersal (mean ± SE of dispersed seeds*Ha^−1^) generated by the different dispersers in the five study sites. TOTAL (mean ± SE) for each disperser is represented by the columns on the left.

**Figure 4:**
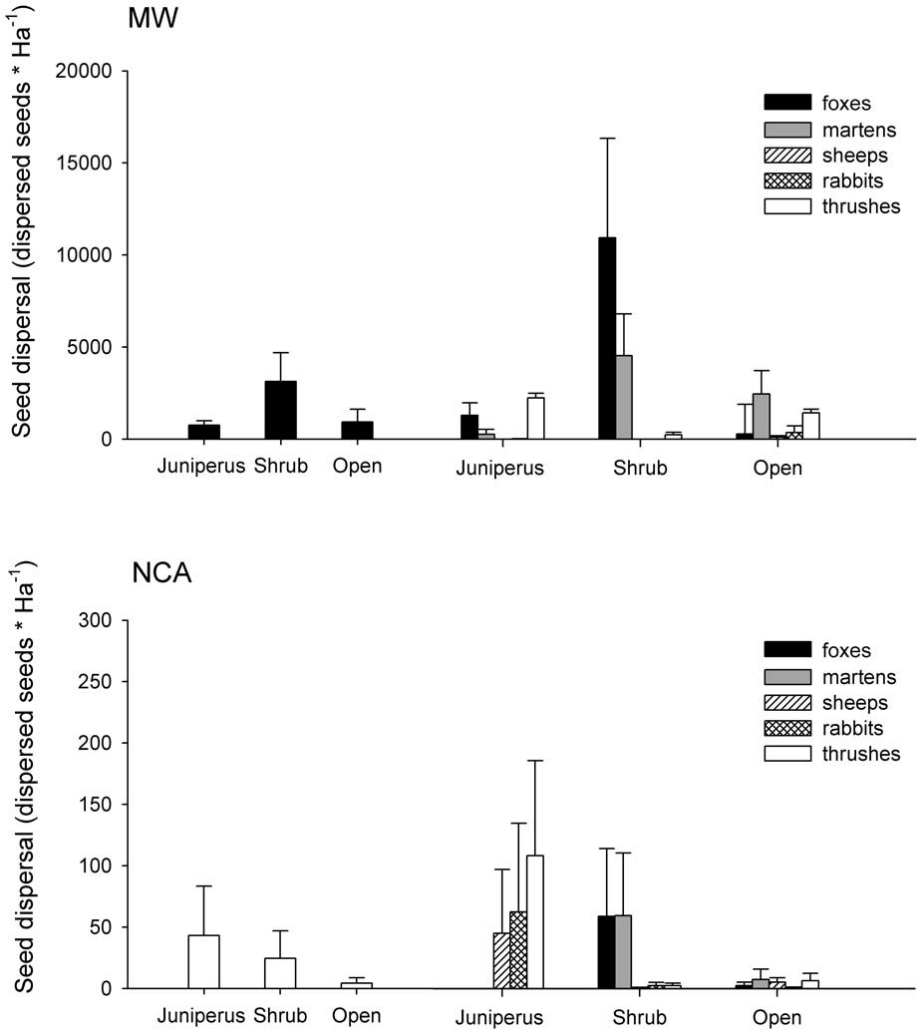
Seed dispersal, habitats, microhabitats and dispersers. Seed dispersal (mean ± SE of dispersed seeds* Ha^−1^) generated by the different dispersers in each microhabitat in Mature Woodland (MW) on the upper panel and New colonization Areas (NCA) on the lower panel. For both panels the first three columns correspond to the mean ± SE total dispersed seeds by all dispersers on each microhabitat. Black columns correspond to Mature Woodland and white columns correspond to New colonization areas.

**Figure 5:**
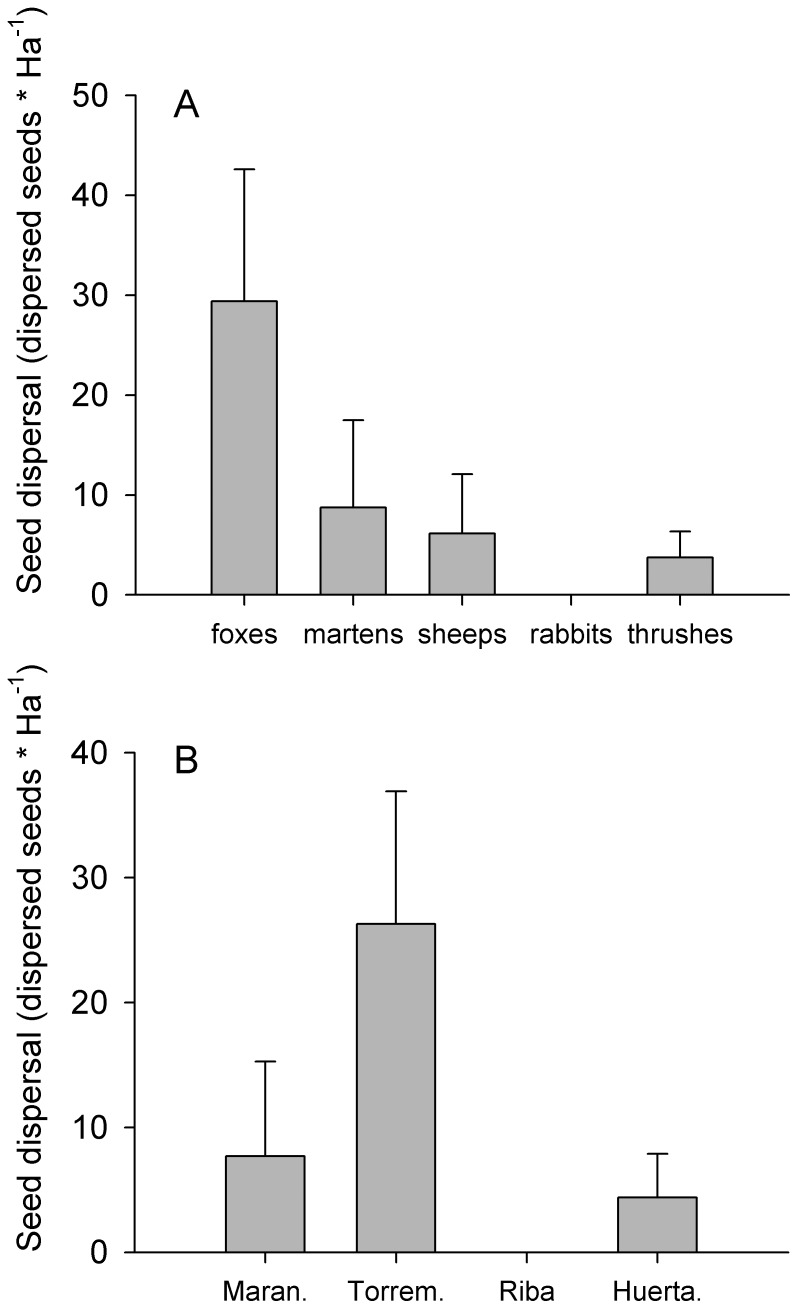
Seed dispersal in agricultural lands. Seed dispersal (mean ± SE of dispersed seeds* Ha^−1^) generated by each disperser species on panel A and on each study site at the panel B.

Seed dispersal was one hundred fold times higher in MW than in NCA. The disperser which produced the highest seed dispersal was the red fox followed by the stone marten, thrushes and lastly herbivores (Table 5, Figure 3). However their relative efficiency was habitat-depend (Figure 3, Figure 4). Carnivores played a more important role on the seed dispersal process in MW while in NCA the relative importance of all dispersers was similar (Figure 3, Figure 4). Seed dispersal did not vary across microhabitats, thus all microhabitats receive a similar amount of seeds as a result of the different deposition patterns of dispersers (Figure 4). Both species of carnivores presented a clear preference for shrub and open microhabitats in MW and in NCA whereas thrushes and herbivores changed their deposition patterns with the habitat. Thrushes dispersed similar quantities of seeds beneath the crown of *J. thurifera* trees and open areas in MW while in NCA most of the seeds dispersed by them where on *J. thurifera* microhabitat. On the other hand herbivores in MW dispersed small number of seeds and mostly in open microhabitats while in NCA they dispersed more seeds and mostly beneath the crown of *J. thurifera* trees (Figure 4). For AL, carnivores were the main dispersers especially foxes (figure 5), which were the only ones with an estimate significantly different from 0 (Estimate 29.37, P = 0.0031).

**Table 5:** General mixed model result for seed dispersal analyses for mature woodland (MW) and new colonization areas (NCA).

Fixed effects		Parameter value	SE	DF	t-value	p-value
Intercept		**−**1088.47	686.86	93	**−**1.58	0.116
Habitat	**Woodland**	**2139.98**	**502.88**	**93**	**4.26**	**<0.001**
Disperser	Stone marten	983.49	676.23	93	1.45	0.149
	Sheep	**−**41.71	676.23	93	**−**0.06	0.951
	**Red fox**	**2160.13**	**676.23**	**93**	**3.19**	**0.002**
	Thrushes	514.63	676.23	93	0.76	0.449
Microhabitat	Shrub	704.36	523.81	93	1.34	0.182
	*J. thurifera* tree	**−**305.48	523.81	93	**−**0.58	0.561
**Random effects**						
	Intercept	Residual				
SD	726.70	2191.24				

Significant effects (P<0.05) are indicated in bold. When P value was smaller than 0.001, <0.001 was indicated. D.F: degrees of freedom. Missing levels of factors (disperser: rabbit; habitat: new colonization areas; microhabitat: open) are included on the intercept.

## Discussion

The main functional groups of dispersers of *J. thurifera* were thrushes and carnivores since herbivores, especially sheeps, dispersed significantly fewer seeds. According to our expectations thrushes and carnivores had a critical role in the process of woodland expansion and colonization of abandoned fields. Both thrushes and carnivores differed in their efficiency according to their feeding behavior and post dispersal pattern, which was modulated by habitat type and particularly by environmental heterogeneity and ecosystem structure. This complex interaction between dispersers and ecosystem heterogeneity surely is conditioning the spatial and genetic structure of the expanding and former woodlands. Our results also shed light on the poorly understood process of seed dispersal in heterogeneous habitats at the landscape scale, especially complex in the case of very fragmented or highly disturbed landscapes such as abandoned fields [36].

### Differential Role of the Disperser Community on the Process of Woodland Expansion

It is well known the role of frugivores birds on plant dispersal far from parental trees [37], [38], since large and medium-sized birds can fly intermediate and long distances (>200 m), even across open areas located between forest or shrubland remnants [16], [39], [40]. In the case of thrushes, some species, as *T. viscivorus*, *T. pilaris and T. iliacus*, showed flight distances ranging from 50 m to 300 m after feeding [16], [17], or even longer (>500 m, personal observation). They perform high-height exploratory flights in large flocks [41] which may contribute to long dispersal events outside mature plant populations on woodland remnants, even in agricultural or abandoned lands as also found in our paper (see [42] for similar north-American landscapes). However the importance of thrushes as disperses in new colonization areas is conditioned to the presence of attractive perches as high trees [17], [43] with big crop size [41], [44]. According to our results a higher percentage cover of other fleshy fruited species was positively related with a higher number of seeds dispersed per faeces. This could be the explanation to the highly different dispersal patterns found for thrushes in two of our study sites, Riba and Huerta. It seems that a more abundant fruiting neighbourhood could produce an attraction effect on thrushes and enhance seed dispersal for the target species even if this one is not very abundant. A similar pattern has previously been shown in other ecological contexts [44], [45]. It seems a general rule that for fleshy fruited species is very positive in dispersal terms to have a heterospecific fruiting neighbourhood. Obviously, depending on total availability of different fruiting species and their spatial location this positive effect on seed dispersal could turn into a competition effect [46].

Regarding carnivores many studies in Mediterranean ecosystems point out their relevance in long-distance seed dispersal due to their wide home range, generalist diet and high retention time in the gut [15], [16], [47], [48]. Seed dispersal produced by carnivores is independent on the current presence of mature fruiting trees [19] as shown also in our study system where we found a higher incidence of red foxes and stone martens as dispersers in active agricultural lands. This means that for primary colonization where no trees of the species are present, carnivores, especially red foxes, could be essential members of the dispersal community and may promote natural restoration of degrade lands as recently proposed [49].

### Differential Role of the Disperser Community on Mature Woodlands

Numerous studies point out that thrushes are the main dispersers of *Juniperus* while the contribution of mammals is occasional and less relevant [24], [25], [50], [51], [52]. Our results do not support such statement, as carnivores and specially red foxes, were by far the main dispersers in two of the tree studied mature woodlands. Changes in tree species cover and structure could produce variations in the availability of different food resources for carnivores. According to our results a higher diversity in tree cover was negatively related with the consumption of *J. thurifera* fruits and with the total number of seeds dispersed per faeces, both variables were notably lower in the mature woodland of Cobeta compared with the other MWs studied. This result could be explained by the existence of a high diversity in the tree layer with the presence of pines and oak species. Higher tree diversity and the presence of some deciduous species promote a more abundant litter cover which has been related with a more abundant and diverse insects and rodents communities [53], [54], [55], [56]. Both, insects and small rodents are consumed and frequently preferred by carnivores [57], [58], [59] due to higher protein content. Therefore we speculate that a higher diversity on the tree layer may had produced a higher availability of trophic resources different from fruits provoking a shift in carnivores diets, More specifically this hypothesis should help to explain the low seed dispersal generated by carnivores in Cobeta [12]. In support to this hypothesis carnivores’ faeces found in the MW of Cobeta contained mainly small-mammals remains (personal observation).

Thrushes‘ population present in the study area were mainly wintering migrants. In their arrival and during their stay they make prospect flights searching for good patches for feeding and avoiding predators [12], [24], [60]. According to our results, thrushes profited fruit resources according to their abundance. In the studied sites where *J. thurifera* cover and crop were abundant, thrushes dispersed a high and similar quantity of seeds. As a result mature woodlands which offered enough crop size, independently of their spatial structure, and presence of other tree species, would result appropriate for wintering thrushes. Thus, as long as migratory period of thrushes and fruiting moment will be accomplished we could assure that thrushes would be constant and faithful dispersers of *J. thurifera*.

### Seed Dispersal and Deposition Pattern Between Microhabitats

As a result of dispersers’ deposition pattern, shrubs and open microhabitat will receive more seeds dispersed by carnivores while *J. thurifera* canopies will receive more seeds dispersed by thrushes. Thrushes and carnivores had a different clumping pattern (1 seed/faeces for thrushes *versus* 50 for carnivores) and we detected higher seeds weight for carnivores than thrushes (unpublished data) which suggests the existence of a playground for evolution to operate. Therefore as a result of dispersers behaviour seeds dispersed on open or shrub microhabitats are heavier than those dispersed beneath the crown of *J. thurifera* tree. Whether or not heavier seeds in open microhabitats will increase or decrease the probability to be predated, secondary dispersed or germinated and finally established remains unknown. Any case recruitment, and species traits’ evolution, would be highly influenced by the interaction among the quantity of seeds do arrive to a microhabitat, the traits selected by dispersers (e.g. seed size) and the environmental characteristics of each microhabitat [61].

### Conclusions

Abandonment of agricultural activities has promoted the colonization of many fields by shrub and tree species. For ecosystems dominated by fleshy fruited species, the arrival of seeds as the first step in the process of colonization is mediated by the feeding behaviour and post dispersal deposition pattern of the community of dispersers as shown here. Our results are congruent with our expectations that the role of carnivores is critical for moving seeds into agricultural lands where isolated trees and perches are absent, therefore this functional group of dispersers are a critical member of the dispersal assemblage for promoting the colonization of abandoned fields. Although, in order to describe the importance of seed dispersal in ecological processes, it is essential to take into account the whole dispersal community together with the environmental heterogeneity occurring at the landscape level, (e.g. vegetation cover and composition), as these variables significantly influence dispersers’ behaviour. Our results showed a decrease in seed dispersal when tree species apart from *J. thurifera* are present, therefore in ecosystems where *J. thurifera* is not the dominant species its dispersal and therefore regeneration and colonization of abandoned fields could be constrained. This finding together with the results described in [62] suggest that *J. thurifera* open woodlands with a reduced grazing pressure, as occurred in our study sties, could produce a shift of their typical open monospecific woodlands towards a closed and more diverse canopy forest. In this scenario seed dispersal of *J. thurifera* could result limited. Therefore, having into account that these formations have conservation concerns the diversity of the dispersal community is an important ecosystem feature that should be preserved and even managed (e.g. avoiding hunting thrushes and predators control of medium carnivores such as red foxes and stone martens) in order to promote the colonization of abandoned fields, the regeneration of former woodlands [63], and the ecosystem services provided by them, such as net gain of value habitat, water and nutrient cycling and carbon sink capacity [23].

## Supporting Information

Table S1 Faeces abundance and number of *J. thurifera* seeds per faeces. MW: mature woodland. NCA: new colonization areas. AL: active agricultural lands. Mh: Microhabitat. N: Number of faeces* ha^−1^. Nj: Percentage of faeces with *J. thurifera* seeds respect the total number of faeces. S/N: average number of *J. thurifera* seeds*faece^−1^.
(DOCX)
