# Supplementary material for: Colonization of Abandoned Land by Juniperus thurifera Is Mediated by the Interaction of a Diverse Dispersal Assemblage and Environmental Heterogeneity
Source: PLoS One. 2012 Oct 10;7(10):e46993. doi: 10.1371/journal.pone.0046993 (PMC3468541; doi:10.1371/journal.pone.0046993)
Supplement: Table S1 — Faeces abundance and number of J. thurifera seeds per faeces. MW: mature woodland. NCA: new colonization areas. AL: active agricultural lands. Mh: Microhabitat. N: Number of faeces* ha−1. Nj: Percentage of faeces with J. thurifera seeds respect the total number of faeces. S/N: average number of J. thurifera seeds*faece−1. (DOCX) [file pone.0046993.s001.docx]

**Supplementary material**

**Table S1. Faeces abundance and number of *J. thurifera* seeds per faeces.**

|  |  |  | Red fox | | | Stone marten | | | Rabbit | | | Sheep | | | Thrushes | | |
| --- | --- | --- | --- | --- | --- | --- | --- | --- | --- | --- | --- | --- | --- | --- | --- | --- | --- |
| Site | Habitat | Mh | N | Nj | S/N | N | Nj | S/N | N | Nj | S/N | N | Nj | S/N | N | Nj | S/N |
| Maranchón | MW | open | 83 | 76 | 69 | 49 | 100 | 84 | 3 | 100 | 11 | 49 | 29 | 1.12 | 2029 | 89 | 0.62 |
|  |  | shrub | 219 | 88 | 81 | 164 | 75 | 41 | 0 | 0 | 0 | 0 | 0 | 0 | 222 | 0 | 0.00 |
|  |  | J. thurifera | 44 | 88 | 53 | 16 | 100 | 49 | 5 | 100 | 5 | 5 | 0 | 0 | 3270 | 90 | 0.63 |
|  | NCA | open | 1 | 0 | 0 | 1 | 0 | 0 | 5 | 0 | 0 | 18 | 9 | 0.68 | 3 | 67 | 0.83 |
|  |  | shrub | 4 | 50 | 19 | 2 | 100 | 27 | 0 | 0 | 0 | 2 |  | 0 | 4 | 50 | 0.50 |
|  |  | J. thurifera | 0 | 0 | 0 | 0 | 0 | 0 | 0 | 0 | 0 | 3 | 0 | 0 | 23 | 71 | 0.57 |
|  | AL | open | 2 | 100 | 25 | 1 | 0 | 0 | 1 | 0 | 0 | 9 | 6 | 0.06 | 1 | 0 | 0.00 |
| Torremocha | MW | open | 50 | 92 | 75 | 42 | 100 | 79 | 29 | 100 | 37 | 104 | 48 | 2.40 | 1971 | 93 | 0.90 |
|  |  | shrub | 208 | 90 | 72 | 146 | 100 | 47 | 0 | 0 | 0 | 0 | 0 | 0 | 444 | 88 | 1.46 |
|  |  | J. thurifera | 11 | 100 | 143 | 0 | 0 | 0 | 0 | 0 | 0 | 4 | 0 | 0 | 3127 | 92 | 0.88 |
|  | NCA | open | 3 | 20 | 4 | 1 | 100 | 58 | 1 | 100 | 4 | 11 | 19 | 0.81 | 21 | 90 | 1.03 |
|  |  | shrub | 9 | 60 | 21 | 4 | 100 | 100 | 4 | 50 | 3 | 7 | 25 | 0.25 | 13 | 43 | 0.82 |
|  |  | J. thurifera | 0 | 0 | 0 | 0 | 0 | 0 | 25 | 60 | 10 | 10 | 50 | 18 | 280 | 96 | 1.05 |
|  | AL | open | 2 | 33 | 41 | 2 | 50 | 35 | 0 | 0 | 0 | 8 | 3 | 33.33 | 35 | 69 | 0.79 |
| Cobeta | MW | open | 29 | 40 | 7 | 11 | 0 | 0 | 0 | 0 | 0 | 155 | 0 | 0 | 1812 | 95 | 0.66 |
|  |  | shrub | 98 | 17 | 3 | 33 | 0 | 0 | 0 | 0 | 0 | 0 | 0 | 0 | 333 | 100 | 0.83 |
|  |  | J. thurifera | 0 | 0 | 0 | 0 | 0 | 0 | 0 | 0 | 0 | 18 | 80 | 0.20 | 2873 | 94 | 0.66 |
| Riba | NCA | open | 12 | 0 | 0 | 2 | 0 | 0 | 0 | 0 | 0 | 7 | 0 | 0 | 12 | 20 | 0.17 |
|  |  | shrub | 3 | 0 | 0 | 0 | 0 | 0 | 0 | 0 | 0 | 0 | 0 | 0 | 0 | 0.00 | 0 |
|  |  | J. thurifera | 0 | 0 | 0 | 50 | 0 | 0 | 0 | 0 | 0 | 17 | 0 | 0 | 317 | 26 | 0.26 |
|  | AL | open | 1 | 0 | 0 | 6 | 0 | 0 | 1 | 0 | 0 | 3 | 0 | 0 | 0 | 0 | 0 |
| Huerta | NCA | open | 0 | 0 | 0 | 0 | 0 | 0 | 2 | 0 | 0 | 11 | 0 | 0 | 4 | 25 | 0.25 |
|  |  | shrub | 0 | 0 | 0 | 0 | 0 | 0 | 0 | 0 | 0 | 0 | 0 | 0 | 0 | 0 | 0.00 |
|  |  | J. thurifera | 0 | 0 | 0 | 0 | 0 | 0 | 0 | 0 | 0 | 10 | 0 | 0 | 10 | 100 | 1 |
|  | AL | open | 1 | 100 | 18 | 2 | 0 | 0 | 0 | 0 | 0 | 5 | 0 | 0 | 4 | 100 | 1.00 |
